# Sex differences in outcomes and associated factors among stroke patients with small artery occlusion in China

**DOI:** 10.1186/s13293-018-0194-6

**Published:** 2018-08-02

**Authors:** Qing Qiao, Yan Hong, Wenjuan Zhao, Guanen Zhou, Qian Liu, Xianjia Ning, Jinghua Wang, Zhongping An

**Affiliations:** 1 Department of Neurology, Tianjin Huanhu Hospital, 6 Jizhao Road, Jinnan District, Tianjin, 300350 China; 2 Tianjin Key Laboratory of Cerebral Vascular and Neurodegenerative Disease, 6 Jizhao Road, Jinnan District, Tianjin, 300350 China; 3 Department of Epidemiology, Tianjin Neurological Institute, 154 Anshan Road, Heping District, Tianjin, 300052 China; 4 Department of Neurology, Tianjin Medical University General Hospital, 154 Anshan Road, Heping District, Tianjin, 300052 China; 5 Tianjin Neurological Institute, Key Laboratory of Post-Neuroinjury Neuro-repair and Regeneration in Central Nervous System, Ministry of Education and Tianjin City, 154 Anshan Road, Heping District, Tianjin, 300052 China

**Keywords:** Sex differences, Small artery occlusion, Subtypes, Outcomes, Risk factors

## Abstract

**Background:**

Sex differences in outcomes after small artery occlusion (SAO) stroke have not been well described, particularly in a Chinese population. We aimed to assess sex differences in outcomes and related risk factors among patients with SAO.

**Methods:**

All consecutive patients with SAO were recruited between May 2005 and September 2014. Clinical features and risk factors were recorded. The mortality, recurrence, and dependency rates at 3 months after stroke were assessed.

**Results:**

A total of 2524 patients with SAO were included in this study. There was a higher frequency of mild stroke, current smoking, and alcohol consumption in men than in women. Women were more likely than men to be older, to have diabetes and obesity, and to have higher total cholesterol, high-density lipoprotein cholesterol, and low-density lipoprotein cholesterol levels. There were worse outcomes in men than in women at 3 months after stroke (*P* < 0.05). There were more independent risk factors of poor outcome in men than in women. Older age was a common predictive factor of outcome both in men and in women. In men, low triglyceride levels and high fasting plasma glucose levels were independent risk factors for mortality; in addition, a high low-density lipoprotein cholesterol level was associated with recurrence. Moreover, in men, moderate and severe stroke, and high total cholesterol and fasting plasma glucose levels were risk factors for dependency. A negative association was found between low-density lipoprotein cholesterol level and risk of mortality and between total cholesterol level and risk of recurrence in women.

**Conclusions:**

These findings suggest that it is crucial to control conventional risk factors and fasting plasma glucose and lipid levels among patients with SAO, especially male patients, to reduce the burden of stroke in China.

## Background

Stroke incidence has declined in developed countries over the past decades, but it has increased in China, becoming the leading cause of death in rural areas and the third leading cause of death in urban areas [1–4]. Furthermore, stroke is now the leading cause of death in China overall, accounting for > 40% of all-cause deaths [5].

Ischemic stroke is characterized by highly differentiated subtypes [6, 7]. Small artery occlusion (SAO) is a major ischemic stroke subtype that occurs more commonly in Asian than Western populations [8, 9]. A higher prevalence of small-vessel occlusion was observed in Chinese stroke patients than in Caucasian stroke patients (33.1% vs. 19.3%) [10].

Stroke has a greater impact on women than on men due to the higher longevity in women [11–15]. However, sex differences in outcomes and relevant risk factors among patients with SAO are unclear. Thus, we aimed to assess differences in outcomes and risk factors according to sex among patients with SAO in a large hospital-based stroke registry in China.

## Methods

### Patient selection

This study used data from a stroke registry from three hospitals in Tianjin, China: Tianjin Medical University General Hospital (a university hospital that primarily admits patients from urban areas and patients transferred from rural areas), Tianjin Huanhu Hospital (a neurological specialized hospital that primarily admits patients from urban and rural areas in Tianjin), and Tianjin Haibin People’s Hospital (an oilfield staff hospital that primarily admits patients working in the Dagang oilfield and their family members).

All acute ischemic stroke patients diagnosed with SAO according to the Trial of Org 10172 in Acute Stroke Treatment (TOAST) classification from May 2005 to September 2014 were included in this study. In this study, patients who died before the imaging examination and patients with transient ischemic attack were excluded. Information on clinical characteristics and outcomes of all patients with ischemic stroke who were admitted to the stroke units in these three hospitals within 72 h after stroke onset was collected.

The study was approved by the ethics committee at Tianjin Huanhu Hospital and was found to conform to the Declaration of Helsinki regarding use of human subjects; written informed consent was obtained from each patient during recruitment.

### SAO diagnosis criteria

SAO was diagnosed according to the following criteria [16]: (1) patients presented with one of the traditional clinical lacunar syndromes and no evidence of cerebralcortical dysfunction; (2) the infarction was located in the subcortex or brainstem and was < 1.5 cm in diameter on computed tomography/magnetic resonance imaging; (3) a previous history of diabetes mellitus (DM) or hypertension supported the clinical diagnosis; (4) potential cardiac embolism was excluded; and (5) < 50% stenosis in an ipsilateral artery was observed.

### Clinical features and risk factors

Clinical features included stroke subtypes, stroke severity, stroke risk factors, neurological function score, and laboratorial values. Stroke subtypes were classified according to Oxfordshire Community Stroke Project (OCSP) criteria on admission; subtypes included total anterior circulation infarct, partial anterior circulation infarct, lacunar infarct, and posterior circulation infarct (POCI) [17]. Stroke severity was categorized into three groups according to National Institutes of Health Stroke Scale (NIHSS) score: mild (NIHSS score ≤ 7), moderate (NIHSS score 8–16), and severe (NIHSS score ≥ 17) [18]. Stroke risk factors included hypertension (defined as systolic blood pressure ≥ 140 mmHg/diastolic blood pressure ≥ 90 mmHg, self-reported previous history of hypertension, or using antihypertension drugs), DM (defined as fasting plasma glucose ≥7 mmol/L, self-reported history of DM, or using hypoglycemic medications at discharge), atrial fibrillation (AF; defined as self-reported history of AF, confirmed by at least one electrocardiogram, or the presence of the arrhythmia during hospitalization), obesity (defined as body mass index ≥ 30 kg/m^2^), current smoking (≥ 1 cigarette per day for more than 1 year), and alcohol consumption (drinking alcohol at least once per week for more than 1 year).

The NIHSS score, modified Rankin Scale (mRS) score, and Barthel Index (BI) were evaluated on admission, at discharge, and at 3 and 12 months after stroke. Moreover, the fasting levels on admission of total cholesterol (TC), triglycerides (TG), high-density lipoprotein cholesterol (HDL-C), low-density lipoprotein cholesterol (LDL-C), and fasting plasma glucose (FPG) were recorded in this study.

### Outcome assessments

Outcomes included mortality, dependency, and recurrence rates 3 months after stroke. Mortality was defined as all-cause cumulative death at the corresponding follow-up time point. Recurrence was defined as all new-onset vascular events, including stroke, myocardial infarction, and venous thrombosis. Dependency was defined as an mRS score > 2 [18]. Follow-up was conducted according to a predetermined procedure; the same trained senior neurologist collected data at 3 months. Follow-up was performed for all patients by face-to-face interview, except for patients who were re-examined in their local hospitals, who completed follow-up by telephone (accounted for 2.1%).

### Management and quality control

This study was a hospital-based follow-up study. There were three groups overseeing the management of all patients into this stroke registry system. The assessment group consisted of five trained senior neurologists who assessed neurological score during hospitalization. The follow-up group consisted of three senior trained neurologists who performed the reexamination, including evaluating neurological scores, managing risk factors, and directing treatment and rehabilitation. The quality control group consisted of the director of the department of neurology, an epidemiologist, and a data management specialist, who conducted a sampled confirmation for 20% of all patients every month.

### Stroke treatment

The most common cause of SAO is hypertension; antihypertensives were prescribed for patients as appropriate. Simultaneously, statins were administered to those with dyslipidemia or evidence of atherosclerosis, and anticoagulants were administered to patients who SAO was caused by AF. Generally, thrombolytic therapy was administered for revascularization within 4 h of stroke, and antithrombotic therapy and treatment for applicable risk factors (such as hypertension, diabetes, dyslipidemia) were given during the acute stage of stroke.

### Statistical analysis

Continuous variables are presented as means with standard deviations (age and TC, TG, HDL-C, LDL-C, and FPG levels) or as medians with interquartile ranges (NIHSS, BI, mRS) and were compared between men and women using Student’s *t* test or the Mann-Whitney *U* test. Categorized variables are presented as numbers of cases (rates) and were compared between men and women using the chi-square test. The factors of recurrence were assessed by multivariate stepwise regression after adjusting for covariates (the *P* value threshold for remaining in the equation was 0.05, while that for removing the covariate from the equation was 0.10); results are presented using adjusted odds ratios (OR) with 95% confidence intervals (CI). All statistical analyses were performed using SPSS version 19.0 (SPSS Inc., Chicago, IL), and a two-tailed *P* value < 0.05 generally indicated statistical significance, but statistical significance in the multivariate analysis was defined as *P* value < 0.05/number of groups. The thresholds of statistical significance were *P* < 0.0125 for OCSP classification and *P* < 0.0167 for stroke severity.

## Results

### Patient selection

**Fig. 1:**
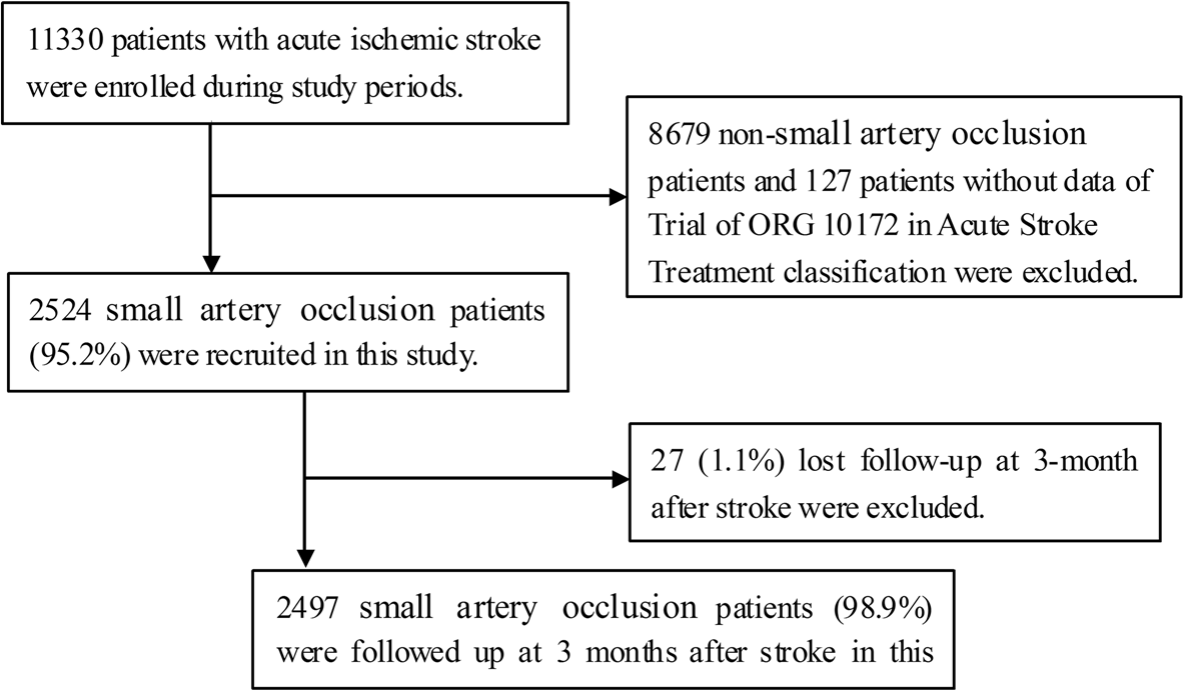
Flow diagram of participants

A total of 11,330 patients with acute ischemic stroke were recruited during the study period, and 2524 patients with SAO were included in this study after excluding 8679 non-SAO patients and 127 patients without TOAST classification data. The response rate was 98.9% 3 months after stroke. Moreover, there were 27 SAO patients (1.1%) lost to follow-up 3 months after stroke. We excluded these patients from the analysis (Fig. 1).

### Sex differences in clinical features among patients with SAO

Of these patients with SAO, there were 1696 men (67.2%) and 828 women (32.8%), with mean ages of 64.10 years overall, 61.93 years for men, and 65.62 years for women (*P* < 0.001). Men were more likely than women to be < 75 years (84.6% vs. 79.6%), have mild stroke (84.1% vs. 79.6%), smoke (50.4% vs. 12.0%), and consume alcohol (27.1% vs. 1.8%; all *P* < 0.001). The prevalence rates of DM and obesity were higher in women than in men. Moreover, women had worse neurological function than men on admission, and average TC, HDL-C, and LDL-levels C were higher in women than in men (Table 1).

**Table 1:** Sex differences in clinical features and risk factors among patients with SAO

Characteristics	Men (*n* = 1696)	Women (*n* = 828)	*t*/*χ*^2^	df	*P*
Age, years, means (SD)	61.93 (11.41)	65.62 (10.86)	7.736	2522	< 0.001
Age group, *n* (%)			34.828	1	< 0.001
< 75 years	1435 (84.6)	620 (74.9)			
≥ 75 years	261 (15.4)	208 (25.1)			
OCSP classification, *n* (%)					
PACI	642 (37.9)	354 (42.8)	5.592	1	ns
TAPI	30 (1.8)	13 (1.6)	0.131	1	ns
LACI	473 (27.9)	220 (26.6)	0.486	1	ns
POCI	551 (32.5)	241 (29.1)	2.955	1	ns
Stroke severity*, *n* (%)					
Mild	1427 (84.1)	656 (79.6)	7.930	1	0.005
Moderate	234 (13.8)	141 (17.1)	4.810	1	ns
Severe	35 (2.1)	27 (3.3)	3.400	1	ns
Risk factors, *n* (%)					
Hypertension	1278 (75.4)	638 (77.1)	0.879	1	ns
Diabetes	514 (30.3)	303 (36.6)	10.048	1	0.002
Atrial fibrillation	41 (2.4)	18 (2.2)	0.145	1	ns
Obesity	139 (8.2)	152 (18.4)	56.325	1	< 0.001
Current smoking	854 (50.4)	99 (12.0)	349.041	1	< 0.001
Alcohol drinking	459 (27.1)	15 (1.8)	232.599	1	< 0.001
Neurological function^†^					
NIHSS	4 (4)	4 (5)	15.928	1	< 0.001
mRS	2 (3)	3 (3)	12.908	1	< 0.001
BI	75 (45)	70 (45)	33.129	1	< 0.001
Laboratory testing, mmol/L, means (SE)					
FPG	6.37 (2.72)	6.58 (2.73)	1.633	1954	ns
TC	4.74 (0.95)	5.23 (1.04)	11.291	2423	< 0.001
TG	1.64 (0.99)	1.71 (0.96)	1.153	2423	ns
HDL-C	1.04 (0.59)	1.15 (0.29)	4.650	2407	< 0.001
LDL-C	2.92 (0.78)	3.15 (0.83)	6.594	2405	< 0.001

*There were 4 female patients for whom NIHSS scores were not obtained

^†^Data presented as medians with interquartile ranges

### Sex differences in outcomes among patients with SAO

As shown in Table 2, there were worse outcomes in men than in women 3 months after stroke, with mortality, recurrence, and dependency rates of 1.9%, 3.8%, and 8.9% in men and 0.6%, 1.7%, and 6.2% in women, respectively (*P* < 0.05).

**Table 2:** Sex differences in outcomes 3 months after stroke among patients with SAO

Outcomes	Men	Women	*t*/*χ*^2^	df	*P*
Mortality, *n* (%)			6.338	1	0.012
Numbers for followed-up	1678	819			
Death case	32 (1.9)	5 (0.6)			
Recurrence, *n* (%)			7.571	1	0.006
Numbers for followed-up	1646	812			
Recurrent case	62 (3.8)	14 (1.7)			
Dependency, *n* (%)			5.469	1	0.019
Numbers for followed-up	1678	819			
Dependency case	150 (8.9)	51 (6.2)			

### Sex differences in factors associated with outcomes among patients with SAO in the multivariate stepwise regression analysis

In the multivariate analysis, the hazard of mortality 3 months after stroke was associated with older age, moderate stroke, low TG level, and elevated FPG level in men. Older age and elevated LDL-C level were independent risk factors for recurrence, with ORs (95% CIs) of 1.04 (1.01, 1.07; *P* = 0.008) for age and 1.80 (1.25, 2.60; *P* = 0.002) for LDL. Moreover, older age, moderate and severe stroke, and high TC and FPG levels were risk factors for dependency 3 months after stroke. In women, older age was a common risk factor of recurrence and dependency 3 months after stroke. Moreover, LDL-C level was associated with mortality 3 months after SAO, and TC level was independently associated with recurrence (Table 3).

**Table 3:** Factors associated with outcomes 3 months after stroke among male and female patients with SAO by multiple stepwise regression analysis

Risk factors	Reference	Mortality				Recurrence				Dependency			
		OR (95%CI)	*χ* ^2^	df	*P*	OR (95%CI)	*χ* ^2^	df	*P*	OR (95%CI)	*χ* ^2^	df	*P*
Men													
Age	–	1.06 (1.02, 1.11)	7.287	1	0.023	1.04 (1.01, 1.07)	6.804	1	0.008	1.05 (1.03, 1.07)	20.397	1	< 0.001
Severity	Mild												
Moderate		4.21 (1.59, 11.18)	8.332	1	0.004	–			–	2.45 (1.48, 4.05)	12.189	1	< 0.001
Severe		2.88 (0.31, 26.91)	0.913	1	0.355	–			–	5.21 (1.68, 16.09)	8.209	1	0.004
Obesity	No									0.39 (0.15, 1.01)	3.727	1	0.054
TC	–	–				–			–	1.33 (1.07, 1.64)	7.574	1	0.009
TG	–	0.27 (0.10, 0.72)	6.894	1	0.009	–			–	–			–
LDL-C	–					1.80 (1.25, 2.60)	9.837	1	0.002				
FPG	–	1.46 (1.27, 1.67)	28.388	1	< 0.001	–			–	1.16 (1.09, 1.24)	19.466	1	< 0.001
Women													
Age	–	–			–	1.06 (1.02, 1.09)	10.539	1	0.001	1.05 (1.02, 1.08)	10.047	1	0.002
TC	–					0.67 (0.46, 0.97)	4.489	1	0.034				
LDL-C	–	0.15 (0.03, 0.78)	5.086	1	0.024								

### Sex differences in clinical features and risk factors among patients with large artery atherothrombosis (LAA)

Table 4 displays the sex differences in clinical features, risk factors, and outcomes 3 months after stroke among patients with LAA. Similar to the findings with SAO, male patients with LAA were more likely than female patients to have mild stroke, smoke, and drink alcohol; female patients with LAA were more likely than male patients to be older and have DM, obesity, poor neurological function, and high TC, HDL-C, and LDL-C levels (all *P* < 0.05). In contrast to the findings with SAO, male patients with LAA were more likely than female patients to have POCI; the frequency of POCI was 37.1% in men and 33.4% in women (*P* = 0.002). Female patients with LAA were more likely than male patients to have severe stroke (12.3% vs. 9.3%; *P* < 0.001), hypertension (77.1% vs. 75.4%), and high FPG and TG levels (all *P* < 0.001). Moreover, there were significant sex differences in dependency rates 3 months after stroke among patients with LAA.

**Table 4:** Sex differences in clinical features and risk factors among patients with LAA

Characteristics	Men (*n* = 1696)	Women (*n* = 828)	*t*/*χ*^2^	df	*P*
Age, years, means (SD)	62.79 (11.52)	67.26 (10.38)	16.348	7598	< 0.001
Age group, *n* (%)			69.098	1	< 0.001
< 75 years	4171 (81.4)	1808 (73.1)			
≥ 75 years	954 (18.6)	667 (26.9)			
OCSP classification, *n* (%)					
PACI	2927 (57.1)	1509 (61.0)	10.220	1	0.001
TAPI	279 (5.4)	127 (5.1)	0.323	1	ns
LACI	20 (0.4)	12 (0.5)	0.356	1	ns
POCI	1899 (37.1)	827 (33.4)	9.611	1	0.002
Stroke severity, *n* (%)					
Mild	3262 (63.7)	1419 (57.3)	28.251	1	< 0.001
Moderate	1386 (27.0)	752 (30.4)	7.342	1	< 0.001
Severe	476 (9.3)	304 (12.3)	16.233	1	< 0.001
Risk factors, *n* (%)					
Hypertension	1278 (75.4)	638 (77.1)	104.860	1	< 0.001
Diabetes	1576 (30.8)	957 (38.7)	47.060	1	< 0.001
Atrial fibrillation	181 (3.5)	133 (5.4)	14.297	1	< 0.001
Obesity	471 (9.2)	508 (20.5)	191.082	1	< 0.001
Current smoking	2704 (52.8)	374 (15.1)	981.771	1	0.002
Alcohol drinking	1448 (28.3)	25 (1.0)	792.793	1	< 0.001
Neurological function, median (interquartile range)					
NIHSS	6 (7)	6 (8)	36.511	1	0.001
mRS	3 (2)	4 (6)	51.413	1	< 0.001
BI	60 (50)	50 (50)	62.025	1	< 0.001
Laboratory value, mmol/L, means (SE)					
FPG	6.50 (2.67)	6.91 (2.86)	5.376	5733	< 0.001
TC	4.76 (1.04)	5.233 (1.15)	21.085	7253	< 0.001
TG	1.58 (1.00)	1.74 (1.27)	5.808	7253	< 0.001
HDL-C	1.02 (0.27)	1.14 (0.31)	16.734	7210	< 0.001
LDL-C	2.96 (0.84)	3.27 (0.91)	13.989	7203	< 0.001
Prognosis at 3 months after stroke, *n* (%)					
Mortality	355 (7.1)	194 (8.0)	2.011	1	ns
Recurrence	194 (4.2)	89 (4.0)	0.107	1	ns
Dependency	753 (15.0)	416 (17.2)	5.604	1	0.018

## Discussion

This is the first report to describe sex differences in outcomes and relevant risk factors among patients with SAO. We demonstrated that mortality, recurrence, and dependency rates 3 months after stroke were significantly higher in men than in women. Older age was a common risk factor of outcomes both in men and in women. Low TG level and high FPG level were independent risk factors for mortality; high LDL-C level were associated with recurrence in men. Moreover, in men, moderate and severe stroke, and high TC and FPG levels were risk factors for dependency. Negative associations were found between LDL-C level and the risk of mortality and between TC level and the risk of recurrence. Inconsistent with the findings for SAO, there were no significant sex differences in mortality and recurrence rates 3 months after stroke among patients with LAA.

The prevalence rates of small-vessel occlusion and internal carotid artery stenosis are high in Asian countries [8]. However, there are limited reports on SAO; moreover, the reported outcomes among patients with SAO have been controversial. Most previous studies demonstrated favorable short-term outcomes among SAO patients [19, 20] and favorable long-term outcomes among elderly SAO patients [17, 21]. Moreover, young patients with SAO experienced low mortality rates over a 5-year follow-up period [22]. In contrast to these studies, a 10-year follow-up study reported a worse prognosis for those with lacunar stroke compared to the general population [23].

Distinct from previous studies, in the present study, we assessed sex differences in clinical features, outcomes, and associated factors among SAO patients. We found that mortality, recurrence, and dependency rates were higher in men than in women. There were poor outcomes in men 3 months after stroke. Simultaneously, lower prevalence rates of DM and obesity were observed in male SAO patients.

A few studies have investigated the association between DM and the long-term prognosis of patients after acute ischemic stroke [24, 25]; however, the evidence for this association remains controversial. A report from China demonstrated that DM independently predicted poor outcomes within 6 months in Chinese patients after acute ischemic stroke [26]. Several studies have reported that DM was associated with a higher stroke mortality rate [27, 28], but this was not observed in other studies [29, 30]. Furthermore, many studies have indicated that DM is an important predictive factor for dependency [28, 31, 32] and recurrence rates [33–35]. However, several studies have shown no remarkable relationship between DM and stroke prognosis [27, 29, 36]. In addition, one study reported that a lower neurological deficit at admission was observed in stroke patients with DM [37]. A recent study indicated that elevated glycosylated hemoglobin level on admission was adversely associated with functional outcomes 3 months after stroke among patients with SAO [38]; both fasting glucose and DM were associated with an increased risk of stroke in patients diagnosed with a minor stroke or transient ischemic attack [39].

In this study, we found no association in men or women between a previous history of DM and stroke outcome; however, FPG level was positively associated with mortality and dependency in male SAO patients. Moreover, while we found a higher prevalence of DM in women than in men, women had lower mortality, recurrence, and dependency rates. The effective management and control of FPG levels in patients with DM can improve outcomes for female stroke patients, and this may have contributed to the lower risk of poor outcomes among female SAO patients in this study.

Furthermore, the relationship between lipid levels and outcomes among SAO patients remains unclear. In a hospital-based study, low serum TC levels were associated with poor functional outcomes in ischemic stroke patients who had received pre-stroke statin treatment, and the short-term and long-term mortality rates were significantly higher in patients with low cholesterol levels [40]. However, our previous findings suggested that there was no significant association between low cholesterol levels and mortality after stroke [41].

In the present study, among male SAO patients, a positive association between TC level and dependency 3 months after stroke was observed, but there was a negative association between TG level and mortality 3 months after stroke. Statins might have a neuroprotective effect [42–44], but the timeliness of statin therapy may explain why the high TC level increased the risk of dependency among SAO patients 3 months after stroke. A long-term follow-up study is needed in the future to explore the impact of TC on outcomes of SAO patients.

There were several limitations in this study. First, this study was conducted in Tianjin, China, and thus represents a limited population. Second, information regarding medication usage before stroke onset was lacking, and this may have affected evaluation of the association between risk factors and outcomes. Finally, all patients were recruited from stroke units; patients with fatal strokes or silent strokes were excluded from this study, and this could have impacted the assessment of outcomes.

## Conclusion

This is the first report to describe sex differences in outcomes and relevant determinants among patients with SAO. We demonstrated that mortality, recurrence, and dependency rates 3 months after stroke were significantly higher in men than in women. Older age, severe stroke, and high FPG level were independent risk factors for mortality and dependency in men. Moreover, in men, TG level was negatively associated with mortality, TC level was positively associated with dependency, and LDL-C level was an independent risk factor for recurrence. A negative association between LDL-C level and mortality was found in women. In addition, older age was positively associated with the risk of recurrence and dependency in women, and TC level was negatively associated with the risk of recurrence. These findings suggest that it is crucial to address the management of FPG and TC levels among patients with SAO, especially male patients, to reduce the burden of stroke in China.

## Abbreviations

BI: Barthel index
CI: Confidence intervals
DM: Diabetes mellitus
FPG: Fasting plasma glucose
HDL-C: High-density lipoprotein cholesterol
LAA: Large artery atherothrombosis
LDL-C: Low-density lipoprotein cholesterol
mRS: Modified Rankin Scale
NIHSS: National Institutes of Health Stroke Scale
OCSP: Oxfordshire community stroke project
OR: Odds ratios
POCI: Posterior circulation infarct
SAO: Small artery occlusion
TC: Total cholesterol
TG: Triglycerides
TOAST: Trial of Org 10172 in acute stroke treatment
